# Cortical spreading depression and familial hemiplegic migraine 2015

**DOI:** 10.1186/1129-2377-16-S1-A20

**Published:** 2015-09-28

**Authors:** Daniela Pietrobon

**Affiliations:** Department of Biomedical Sciences, University of Padua, Padua, Italy

The molecular and cellular mechanisms of the primary brain dysfunction leading to the onset of a migraine attack and to susceptibility to cortical spreading depression (CSD), the neurophysiological correlate of migraine aura and a likely trigger of the headache mechanisms, remain largely unknown and major open issues in the neurobiology of migraine. Our approach to these open questions is the study of the functional consequences of mutations causing familial hemiplegic migraine type 1 and type 2 (FHM1 and FHM2). FHM1 is caused by gain-of-function mutations in the neuronal CaV2.1 channel, a voltage-gated calcium channel that plays a dominant role in controlling neurotransmitter release at brain excitatory and inhibitory synapses. FHM2 is caused by loss-of-function mutations in the glial alpha2 Na/K-ATPase, an isoform that is thought to have specific roles in K+ and glutamate clearance by astrocytes and in astrocyte Ca2+ homeostasis. Knockin (KI) mouse models carrying FHM1 or FHM2 mutations show a lower threshold for CSD induction and a higher velocity of CSD propagation. We have investigated the cortical mechanisms underlying the facilitation of experimental CSD in FHM1 and FHM2 KI mice by studying synaptic transmission at cortical excitatory and inhibitory synapses and the rate of glutamate and K+ clearance by cortical astrocytes in acute cortical slices. Our findings are consistent with the conclusion that increased activation of NMDA receptors due to enhanced cortical glutammatergic synaptic transmission in FHM1 and to reduced rate of glutamate clearance at cortical excitatory synapses in FHM2 contributes to the facilitation of CSD in FHM KI mice. The data from FHM mouse models support the view of migraine as a disorder of brain excitability characterized by dysregulation of the excitatory-inhibitory E/I balance, and point to episodic disruption of the E/I balance and neuronal hyperactivity due to excessive recurrent glutammatergic transmission as the basis for vulnerability to CSD ignition in FHM.

